# Anthropogenic Infection of Cats during the 2020 COVID-19 Pandemic

**DOI:** 10.3390/v13020185

**Published:** 2021-01-26

**Authors:** Margaret J. Hosie, Regina Hofmann-Lehmann, Katrin Hartmann, Herman Egberink, Uwe Truyen, Diane D. Addie, Sándor Belák, Corine Boucraut-Baralon, Tadeusz Frymus, Albert Lloret, Hans Lutz, Fulvio Marsilio, Maria Grazia Pennisi, Séverine Tasker, Etienne Thiry, Karin Möstl

**Affiliations:** 1 MRC—University of Glasgow Centre for Virus Research, Glasgow G61 1QH, UK; Margaret.Hosie@glasgow.ac.uk; 2 Clinical Laboratory, Department of Clinical Diagnostics and Services, Center for Clinical Studies, Vetsuisse Faculty, University of Zurich, 8057 Zurich, Switzerland; Hans.Lutz@uzh.ch; 3 Clinic of Small Animal Medicine, Centre for Clinical Veterinary Medicine, LMU Munich, 80539 Munich, Germany; hartmann@lmu.de; 4 Department of Biomolecular Health Sciences, Faculty of Veterinary Medicine, University of Utrecht, 3584 CL Utrecht, The Netherlands; H.F.Egberink@uu.nl; 5 Institute of Animal Hygiene and Veterinary Public Health, University of Leipzig, 04103 Leipzig, Germany; truyen@vetmed.uni-leipzig.de; 6 Maison Zabal, 64470 Etchebar, France; draddie@catvirus.com; 7 Department of Biomedical Sciences and Veterinary Public Health (BVF), Swedish University of Agricultural Sciences (SLU), Box 7036, 750 07 Uppsala, Sweden; sandor.belak@slu.se; 8 Scanelis Laboratory, 31770 Colomiers, France; corine.boucraut@scanelis.com; 9 Department of Small Animal Diseases with Clinic, Institute of Veterinary Medicine, Warsaw University of Life Sciences—SGGW, 02-787 Warsaw, Poland; tadeusz_frymus@sggw.edu.pl; 10 Fundació Hospital Clínic Veterinari, Universitat Autònoma de Barcelona, Bellaterra, 08193 Barcelona, Spain; albert.lloret@uab.es; 11 Faculty of Veterinary Medicine, Università degli Studi di Teramo, 64100 Teramo, Italy; fmarsilio@unite.it; 12 Dipartimento di Scienze Veterinarie, Università di Messina, 98168 Messina, Italy; mariagrazia.pennisi@unime.it; 13 Bristol Veterinary School, University of Bristol, Bristol BS40 5DU, UK; s.tasker@bristol.ac.uk; 14 Linnaeus Group, Shirley, Solihull B90 4BN, UK; 15 Veterinary Virology and Animal Viral Diseases, Department of Infectious and Parasitic Diseases, FARAH Research Centre, Faculty of Veterinary Medicine, Liège University, B-4000 Liège, Belgium; Etienne.Thiry@ulg.ac.be; 16 Institute of Virology, Department for Pathobiology, University of Veterinary Medicine, 1210 Vienna, Austria; karinmoestl@gmail.com

**Keywords:** SARS-CoV-2, COVID-19, domestic cats, wild felids, companion animals, minks, experimental infection, human-to-feline transmission, one health

## Abstract

COVID-19 is a severe acute respiratory syndrome (SARS) caused by a new coronavirus (CoV), SARS-CoV-2, which is closely related to SARS-CoV that jumped the animal–human species barrier and caused a disease outbreak in 2003. SARS-CoV-2 is a betacoronavirus that was first described in 2019, unrelated to the commonly occurring feline coronavirus (FCoV) that is an alphacoronavirus associated with feline infectious peritonitis (FIP). SARS-CoV-2 is highly contagious and has spread globally within a few months, resulting in the current pandemic. Felids have been shown to be susceptible to SARS-CoV-2 infection. Particularly in the Western world, many people live in very close contact with their pet cats, and natural infections of cats in COVID-19-positive households have been described in several countries. In this review, the European Advisory Board on Cat Diseases (ABCD), a scientifically independent board of experts in feline medicine from 11 European Countries, discusses the current status of SARS-CoV infections in cats. The review examines the host range of SARS-CoV-2 and human-to-animal transmissions, including infections in domestic and non-domestic felids, as well as mink-to-human/-cat transmission. It summarises current data on SARS-CoV-2 prevalence in domestic cats and the results of experimental infections of cats and provides expert opinions on the clinical relevance and prevention of SARS-CoV-2 infection in cats.

## 1. Introduction

The coronavirus (CoV) that causes coronavirus disease 2019 (COVID-19) was first isolated in December 2019, in Wuhan City, Hubei province, China. The new virus is closely related to the severe acute respiratory syndrome coronavirus (SARS-CoV) that caused a disease outbreak in 2003 and has been named SARS coronavirus 2 (SARS-CoV-2). SARS-CoV-2 is a member of the genus betacoronavirus, family Coronaviridae, order Nidovirales (Table 1). It is a new strain that has not previously been identified in humans or animals. SARS-CoV-2 did not emerge from any companion animal CoV; neither is it related to the commonly occurring feline coronavirus (FCoV) associated with feline infectious peritonitis. SARS-CoV-2 infection has spread to many countries worldwide, leading to the declaration of a pandemic by the World Health Organisation on 11 March 2020 [1].

## 2. Other Human Coronaviruses

To date, seven human coronaviruses (HCoVs) have been identified [2,3], as shown in Table 1. All can cause respiratory illnesses in humans, ranging in severity from asymptomatic infection or a mild, common cold to pneumonia and bronchiolitis.

In the last two decades, there have been two major human disease outbreaks associated with coronaviruses: SARS [4] and Middle Eastern respiratory syndrome (MERS) [5]. Both the SARS and MERS viruses evolved from viruses circulating in bats, the natural reservoir host of many CoV [6,7]. Viruses with highly similar genetic sequences to SARS-CoV-2 have been isolated from bats, which indicates that, similar to previous CoV outbreaks, bats are a potential source of the new CoV. It is currently not clear whether transmission of SARS-CoV-2 occurred directly from bats to humans, or whether transmission occurred indirectly, via an intermediate host.

Three of these seven human coronaviruses (agents of MERS, SARS, and COVID-19) can cause severe illnesses and death, although some infections in some individuals can be mild or asymptomatic. The other four common human coronaviruses typically cause only mild respiratory illnesses in healthy human adults. However, they contribute to a third of common cold infections and can cause life-threatening illnesses in immunocompromised people.

## 3. SARS-CoV Infections in Cats

SARS was first detected in 2002 and originated in a seafood market in Guangdong, China [4,8,9]. The causative agent, SARS-CoV, spread from China around the world [10,11,12], but the outbreak was contained after approximately nine months following strict infection control measures [10]. The epidemic resulted in 8096 reported human cases and 774 deaths in 27 countries [12].

SARS-CoV has been shown to infect a wide range of species under experimental conditions, including masked palm civets [13], monkeys [14,15,16,17,18,19,20], mice [5,21], pigs, chickens [22], guinea pigs [23,24], and Golden Syrian Hamsters [3,25]. The prevalence of SARS-CoV infection in masked palm civets (*Paguma larvata*) that were raised for human consumption was high [26,27], and the animals developed neutralizing antibodies. Masked palm civets were considered to be transient accidental hosts [28,29] and were also confirmed as occasional direct sources of human infections [26,30,31].

Domestic cats can be infected experimentally with SARS-CoV and develop active infection, shedding, and pulmonary changes very similar to those seen in fatal human cases [32]. In an experimental study, SARS-CoV from a human patient who had died from SARS was inoculated intratracheally in cats, and the virus could be isolated from pharyngeal swabs taken on days two to eight post-infection and from nasal swabs taken on days four and six post-infection, but SARS-CoV was not detected in rectal swabs; however, no clinical signs were observed following experimental infection. Four cats were euthanised and necropsied at four days post-infection, and SARS-CoV could be isolated from the trachea and lungs, confirming infection of the lower respiratory tract. All of the SARS-CoV-infected cats that were not euthanised developed neutralising antibodies by day 28 post-infection. Two uninfected cats that were housed together with SARS-CoV-infected cats tested positive for SARS-CoV by RT-PCR, with viral loads gradually increasing from two days post-infection onwards, peaking at days six to eight post-infection. Two in-contact cats showed no clinical signs but developed antibodies by day 28 [32]. In another study in which cats were experimentally infected intratracheally, pulmonary lesions were also described [33]. Histologically, SARS-CoV was detected in cells expressing the ACE-2 receptor, and the cats had developed diffuse alveolar damage and many pathological changes also seen in human SARS patients [33] with the difference that the cats developed also tracheo-bronchoadenitis, which has not been reported in humans [33,34]. The data from the two studies demonstrated that domestic cats are susceptible to experimental infection with SARS-CoV, that the virus could be transmitted to other cats, and that clinical signs and pathology were similar to those in humans [32,33].

Natural infection in field cats was also described during the first SARS outbreak [27,28,31,35,36]. Domestic cats living in an apartment block in Hong Kong where 100 people contracted SARS-CoV infection also tested RT-PCR-positive. However, final proof of direct transmission from humans to cats was lacking [32,37,38]. Oropharyngeal and rectal swabs were collected from cats from a multiple cat household and from two dogs in this apartment block over 14 days, after their owners were diagnosed with SARS; eight cats and one of the dogs tested RT-PCR-positive. Spontaneous infection of cats from another three multiple pet households was demonstrated by RT-PCR of oropharyngeal and rectal swabs collected over a 14-day period [37]. SARS-CoV was also isolated from the cats, with the sequenced virus being indistinguishable from the human isolates [37]. Antibody testing using serum neutralisation assays confirmed SARS-CoV infection in one RT-PCR-positive cat from one household and in four of five cats (including three RT-PCR-positive cats) from another household. The cats were isolated and kept in household groups in single cages and in separate rooms while in isolation. There was limited evidence of spread in the isolation cages (cats in close direct contact with these cats remained uninfected) [32,37,38].

## 4. Host Range of SARS-CoV-2

The host range of a virus depends on several factors. The first step of viral infection occurs when the virus particle binds to a susceptible host cell via specific interactions between the receptor binding site on a viral protein and the virus receptor molecules on the host cell, a key determinant of the host range and tissue tropism of a virus.

Both SARS-CoV and SARS-CoV-2 utilise the angiotensin-converting enzyme 2 (ACE-2) molecule, a single-pass type I membrane protein, as the virus receptor for infection. ACE-2 is highly expressed in the lungs, arteries, heart, kidney, and intestines; it is an important protein involved in blood pressure regulation. In addition to ACE-2, neuropilin-1 facilitates SARS-CoV-2 cell entry and provides a possible pathway into the central nervous system.

The viral receptor binding site lies in a domain on the spike protein (S), a glycoprotein that protrudes from the surface of the virus. The S protein and the viral receptor binding site have been well studied for SARS-CoV, and recently extensive sequence analysis studies and functional tests have been performed with SARS-CoV-2. The sequence of human SARS-CoV-2 is similar to that of CoVs circulating in bats [39] that gave rise to SARS in 2002. Related viruses have been found in Malayan pangolins [40,41], indicating these imported animals might be intermediate hosts, although this has yet to be proven.

Three short regions of the ACE-2 molecule (3 to 11 amino acids long) have been identified that are involved in virus binding, and a comparative analysis of the sequences from different mammals, including humans, apes, macaques, horse, swine, goat, sheep, bovine, cat, dog, rat, mouse, ferret, bat, and civet, revealed some differences [42]. The sequences were identical for all apes, monkeys, and humans, but differences were found in residues that are considered important for virus binding in other species. For cats and dogs, one residue in ACE-2 that is critical for virus binding was different and, most interestingly, the bat and civet sequences contained two critical residues that differed from the human sequence. Although differences in the ACE-2 sequences from different animals have been identified, the impact of these single amino acid changes on receptor binding and the susceptibility of other species to infection is not yet known. However, the high overall sequence identity could explain the relatively broad host range of SARS coronaviruses.

## 5. Evidence of Human-to-Cat Transmission of SARS-CoV-2

At the time of writing (30 December 2020), there have been numerous sporadic reports of domestic animals from COVID-19 households that tested positive for SARS-CoV-2 and were presumed to be infected from their owners. Cats infected with SARS-CoV-2 have been identified in Belgium [43], Hong Kong [44], US [45], France [46], Spain [47,48], Germany [49], UK [50], Italy [51], and Switzerland [52], and these cases, as well as cases from Russia, Denmark, Sweden, Chile, Japan, Brazil, and Argentina have been reported to OIE and appear on a list that is regularly updated by OIE [53].

The first cat that tested positive in Hong Kong was a domestic short-haired cat that was quarantined when the owner was confirmed with COVID-19. Swabs collected from the oral and nasal cavities and the rectum tested positive for SARS-CoV-2 RNA. The cat did not show any signs of disease.

The first reported case in Europe was a cat living in Belgium with its owner, who was self-isolating after testing positive for SARS-CoV-2, that developed clinical signs one week after the owner’s return from Italy. The cat’s clinical signs (anorexia, diarrhoea, vomiting, cough, and shallow breathing) were compatible with a CoV infection (respiratory and/or digestive), and the cat tested positive for the SARS-CoV-2 RNA in successive samples of faeces and vomitus. Positive RT-PCR results were confirmed by sequencing. Nine days after the onset of clinical signs, the cat’s condition improved [43]. The test results were consistent with a high number of viral RNA genomic copies (D. Desmecht, personal communication), indicative of infection following human-to-cat transmission.

The first two cases of feline SARS-CoV-2 infection in the United States were confirmed in New York by the CDC and the U.S. Department of Agriculture (USDA). The cats originated from separate households and were epidemiologically linked to suspected or confirmed human COVID-19 cases in their respective households [54].

In addition to human-to-cat transmission, there have been reports of suspected human-to-mink transmission in farmed mink in the Netherlands [55,56,57], Denmark, Italy, Spain, USA, Sweden, Greece, France, Poland, and Lithuania [58]. It was reported that COVID-19 symptoms were present in humans working on the Dutch farms before clinical signs were observed in mink, and infection was confirmed in one hospitalised person [59]. It is thought that the widespread infection amongst the mink followed the introduction of the virus by humans and subsequent transmission between the mink.

## 6. Evidence of Mink-to-Cat and Mink-to-Human Transmission

At the first mink farm found to be infected with SARS-CoV-2, antibodies against the virus were detected in three of eleven farm cats, but no virus was isolated [55]. Later, farm cats living on all four infected farms were sampled, and seven of 24 cats tested antibody-positive; one cat tested positive for SARS-CoV-2 RNA.

The first two cases of mink-to-human transmission were reported from the Netherlands on two different mink farms. These findings were based on phylogenetic analysis of sequences of the viruses in the infected mink and humans [59,60] and are consistent with possible exposure of farm workers to virus in the environment that had been excreted by the infected mink. Subsequently, it was decided to cull all mink in the affected farms [56,61]. Denmark reported 214 human COVID-19 cases infected with SARS-CoV-2 virus variants related to mink, as well as infected mink at more than 200 mink farms [62]. The SARS-CoV-2 variants that were detected contained three amino acid substitutions and one deletion in the spike (S) protein. Since the S protein contains the receptor-binding domain and is a major target for the immune response, the emergence of these variants has raised concerns that such mutations could, in theory, influence the transmissibility and antigenicity of the virus, and further investigations and studies are ongoing.

## 7. Prevalence of SARS-CoV-2 Infection in Domestic Animals

In Hong Kong, cats and dogs from households with infected people have been quarantined by the Hong Kong Agriculture, Fisheries and Conservation Department (AFCD) [63]. The AFCD conducted tests on these cats and dogs from households with confirmed human COVID-19 cases or persons in close contact with confirmed patients. One of 17 cats [64] and two of 15 dogs [65] from confirmed COVID-19 households that had been placed in quarantine in Hong Kong have tested RT-PCR-positive for SARS-CoV-2 RNA; antibodies were also detected in both of the dogs [66], and the viral sequences from the dogs were identical to those in the respective human contacts. More recently, Barrs et al. [44] reported the results of testing 50 quarantined cats from COVID-19 households or close contacts for SARS-CoV-2 RNA in respiratory and faecal swabs; six healthy cats tested positive, suggesting human-to-feline transmission, and viral genomes sequenced from one cat and its owner were identical.

Zhang et al. [67] reported that cats become infected with SARS-CoV-2 following natural exposure to infected people, with 15 of 102 (14.7%) cat sera collected following the outbreak in Wuhan testing positive for antibodies that recognised the receptor binding domain of SARS-CoV-2 by ELISA, eleven of which (10.8%) also tested positive for neutralising antibodies. The results of this study imply that cat populations could become infected in any region affected by the COVID-19 pandemic. The authors noted that sera from three cats known to be owned by COVID-19 patients and, therefore, presumed to have close contact, demonstrated higher neutralising antibody titres compared to sera collected from either hospitalised or stray cats. Although the mechanism of transmission to stray cats is not fully understood, stray cats could have become infected via SARS-CoV-2 contamination of their environment, from COVID-19 patients who fed the cats, or even as a result of cat-to-cat transmission.

A survey of cats and dogs from confirmed COVID-19 households in France reported a high seroprevalence of SARS-CoV-2 antibodies, ranging from 21–53%, depending on the criteria used to define a positive result [68]. In a longitudinal study of 76 cats and dogs living in 39 COVID-19 households in Texas, US, eight of 17 cats and nine of 59 dogs tested positive for SARS-CoV-2 RNA or neutralising antibodies [69]. Virus was isolated from one out of three RT-PCR positive cats [69].

Other epidemiological studies have been conducted that included samples collected from animals presenting for routine veterinary visits, where there was no known exposure to people infected with SARS-CoV-2. A study of 919 companion animals in Northern Italy that were sampled at a time of frequent human infection reported that 3.3% of dogs and 5.8% of cats displayed titres of neutralising antibodies [70]. In this study, 69 samples for serology originated from dogs (47) and cats (22) from a known COVID-19 affected household and 12.8% (6/47) of these dogs and 4.5% (1/22) of these cats tested positive for SARS-CoV-2-neutralizing antibodies; in addition, the seroprevalence found in dogs from a known COVID-19 positive household was significantly higher than dogs from COVID-19 negative households (1.5%; 2/133) [70]. Along these lines, a German study of 920 serum samples (which had been collected from domestic cats between April and September 2020 for haematological testing) demonstrated that only 0.69% (6/920) of samples contained antibodies against SARS-CoV-2; two of the positive sera had neutralising antibodies [71], suggesting that human-to-cat transmission might be relatively infrequent. Furthermore, a study conducted in a veterinary community of 20 students, two of whom tested positive for COVID-19 and eleven of the remaining 18 displayed symptoms of COVID-19, demonstrated that none of the nine cats and none of the twelve dogs living in the community tested positive by RT-PCR and none of the cats or dogs developed antibodies [72], which confirms that human-to-domestic animal transmission is variable and suggests that transmission is likely minimised where good hygiene is practised.

## 8. Evidence of Human-to-Non-Domestic Cat Transmission of SARS-CoV-2

SARS-CoV-2 infection of a non-domestic cat was first reported by the OIE on 4 April 2020 [73]. Nasal and oropharyngeal swabs and tracheal wash samples collected from a 4-year-old female Malayan tiger (*Panthera tigris jacksoni*) with respiratory signs tested positive for SARS-CoV-2 RNA. The tiger was kept in the Wildlife Conservation Society’s (WCS) Bronx Zoo, where two Malayan tigers, two Amur tigers (*Pantheratigris altaica*), and three African lions (*Panthera leo*) had developed respiratory signs over the course of a week and showed clinical improvement following supportive treatment. On 15 April 2020, one of the three lions was confirmed positive for SARS-CoV-2 [74]. As there had been no new animal introductions for several years, it was presumed that SARS-CoV-2 was transmitted to the tiger from a SARS-CoV-2-infected keeper who was either asymptomatic or pre-symptomatic at the time of exposure. Subsequently, all of the tigers and lions in the group tested positive when faecal samples were examined [75]. In addition, two Malayan tigers in a zoo in Tennessee were tested for SARS-CoV-2 infection when the animals displayed mild coughing, lethargy, and decreased appetite; one of the tigers tested positive [76].

## 9. Experimental Infection of Cats with SARS-CoV-2

It has been shown that cats, ferrets, and Golden Syrian hamsters can be infected experimentally with SARS-CoV-2 [77,78,79]. Shi et al. [77] demonstrated that cats, ferrets, and (to a lesser extent) dogs were susceptible to infection, but not pigs, chickens, or ducks. When 8-month-old domestic cats were infected intranasally with 10^5^ PFU of SARS-CoV-2 isolated from a human patient, viral RNA was detected in the upper respiratory tract, small intestine, and faeces; infectious virus was found only in the upper respiratory tract, but not from the tissues tested. When the same high dose of the virus was used to infect 10-to-14-week-old kittens, viral RNA and infectious virus was detected in the upper respiratory tract, lung, small intestine, and nasal washes, and histopathological changes were observed in the lungs, suggesting that SARS-CoV-2 replicates more efficiently in younger cats. One of three 10-to-14-week-old kittens died on day three after virus exposure. Three infected 10-to-14-week-old kittens and three infected 8-month-old domestic cats were housed individually in cages adjacent to uninfected cats. Subsequently, two animals that were in cages adjacent to experimentally infected cats became infected and developed antibodies; the exposed cats that became infected were one 10-to-14-week-old kitten and one 8-month-old domestic cat. However, as the exposed cats were in cages adjacent to the infected cats within an isolator, it was not clear whether the mode of transmission was via respiratory droplets or faeces, as the exposed cats might have also been exposed to the virus in the faeces of the infected cats.

In another experimental study, three cats were inoculated with SARS-CoV-2 on day 0 and then cohoused, in pairs, with uninfected cats starting one day after inoculation [80]. The inoculated cats shed infectious virus in nasal swabs from day one to three, until day six, and the in-contact cats started shedding virus from day three to day five. No virus was detected in rectal swabs, and none of the cats displayed clinical signs, although all cats developed antibodies confirming infection.

These experimental studies showed that cats are susceptible to SARS-CoV-2 infection [81]; however, the findings suggested that cats are unlikely to develop clinical disease under these experimental conditions. In addition, these studies demonstrated that cats develop a robust neutralising antibody response that prevents cats from being re-infected following a second viral challenge. Further studies will be required to determine how easily SARS-CoV-2 can be transmitted between cats. At present, no transmission event from cats to humans has been reported. 

## 10. Stability of Coronaviruses

CoV are enveloped viruses, and once the envelope is damaged or destroyed, the virus is no longer infectious, which is why handwashing for at least 20 s (the WHO recommends even 40 s [82]) with soap and water can prevent transmission of SARS-CoV-2. However, CoV appear to be more stable in dry conditions compared to many other enveloped viruses, remaining infectious for longer periods of time on surfaces. In addition, extraneous proteins in blood or faeces can protect viruses from inactivation, prolonging viral infectivity [83].

The stability of coronaviruses is variable on surfaces, with the SARS-CoV, a betacoronavirus, being slightly more stable than the alphacoronavirus human coronavirus HCoV-229E [84]. A recent study compared the stability of SARS-CoV and SARS-CoV-2 in aerosols and on surfaces and found virtually identical results [85], with both viruses remaining infectious on dry surfaces for up to 72 h.

The nature of the surfaces, however, is crucial, and SARS-CoV-2 remains infectious for longer on plastic and stainless-steel compared to cardboard or copper surfaces (24–72 h versus 8–24 h, respectively) [85]. For SARS-, MERS-, and other human CoV, persistence was tested on different types of inanimate surfaces (summarized in [86]): at room temperature, persistence of several days was documented on metal, wood, paper, glass, and plastic with a maximum of nine days on plastic in one study [84]. SARS-CoV and SARS-CoV-2 remained viable and infectious in aerosols for hours and on different surfaces for days; these results indicate that aerosol as well as fomite transmission of these viruses can be expected [85].

As with all other known enveloped viruses, CoV are highly susceptible to common chemical disinfectants and are readily inactivated by, e.g., alcohols, household bleach, benzalkonium, aldehydes, and others [86,87]. Differences have been observed between cat litters concerning inactivation of the alphacoronavirus, FCoV. One study revealed that some cat litters, particularly those based on bentonite, can bind and might inactivate CoV shed in faeces and could help to reduce the FCoV load within infected households [88].

It is possible that cats could act as fomites when living in households with COVID-19 owners, although no studies have been published documenting the survival of SARS-CoV-2 on fur. Cats themselves should not be disinfected under any circumstances, as toxicity and burns can result from the inappropriate use of disinfectants that could be ingested during self-grooming.

## 11. Diagnosis

At present, it is recommended that cats should be tested for SARS-CoV-2 infection only following consultation with the appropriate public health authority, since recommendations are different between countries. Testing is available in veterinary laboratories in several European countries, using RT-PCR to detect viral RNA in swabs and ELISA to detect antibodies in serum or plasma as well as virus neutralising antibody tests. Particularly, antibody testing using ELISA should also undergo further evaluation to determine specificity, sensitivity, and potential cross-reactivity for feline samples. Virus isolation from swabs is restricted to specialist laboratories with containment level 3 facilities, as the isolation of SARS-CoV-2 poses a risk to laboratory staff.

## 12. Conclusions

Given the potential for infected owners to transmit the virus to their pets, and the possibility that cats could act as fomites, close contact with cats (and dogs) should be avoided in households where people are infected with SARS-CoV-2 or have symptoms of COVID-19. A One Health approach should be fostered, with Public Health and Veterinary Services sharing information and investigating situations where a person who is infected with SARS-CoV-2 reports being in contact with companion or other animals. If an owner with COVID-19 must continue to care for their pet while ill, they should maintain basic hygiene measures. Such measures include handling animals only when wearing a mask, washing their hands with soap and water for at least 20 s before and after being near or handling their animals, their food, or their supplies, as well as avoiding kissing their pets or sharing food, towels, or the bed with them.

ABCD emphasises that at the time of writing, no transmission event from cats to humans has been reported, although it is recognised that (for ethical reasons and limitations on study design) it is unlikely that direct evidence of cat-to-human transmission could be obtained [89]. In light of recent reports of SARS-CoV-2 infections in some cats living in households with SARS-CoV-2-infected people, as well as the non-domestic cats that were presumed to be infected by their zookeepers, it is recommended that cats from SARS-CoV-2-infected households are kept indoors, until there is a better understanding of how efficiently the virus is transmitted from humans to cats, whether cats can transmit virus to other cats under natural conditions, and whether the virus could be transmitted from cats to humans. Any cat from a COVID-19 household should not be taken into another household. The American Veterinary Medical Association (AVMA) has developed detailed protocols that could be implemented to protect staff when they are exposed to high-risk situations, such as when entering an infected person’s home or coming into proximity with a sick person. AVMA recommends that procedures should be consistent with the most up-to-date guidance from the relevant public health authorities (AVMA, 2020).

This guideline will continue to be updated regularly on the ABCD homepage (www.abcdcatsvets.org) as new data become available. In addition, ABCD has given answers to common questions (see Appendix A); these are also available on the ABCD homepage. Pet owners should always maintain good hygiene practices and under no circumstances should cats be abandoned.

## Figures and Tables

**Table 1:** Classification of coronaviruses (CoVs).

Host	Alpha	Beta	Gamma	Delta
Human	Human CoV-229E, human CoV-NL63	Human CoV-OC43, human CoV- HKU1		
Bat origin (outbreaks in humans)		SARS-CoV-2, SARS-CoV, Middle-Eastern-respiratory-syndrome-related CoV		
Felids	Feline CoV			
Canines	Canine enteric CoV	Canine respiratory CoV		
Porcines	Porcine epidemic diarrhoea, porcine respiratory CoV, transmissible gastroenteritis virus	Porcine haemagglutinating encephalomyelitis virus		Porcine CoV HKU15
Ruminants		Bovine CoV, Antelope CoV, Giraffe CoV		
Equids		Equine CoV		
Bat	Various bat CoVs	Three bat CoVs		
Avian			Turkey CoV, infectious bronchitis virus	Nine avian CoVs
Various		Hedgehog CoV HKU31, Pangolin CoV	Beluga whale CoV-SW1	

## Appendix A

**Common questions on COVID-19 and cats**

Note: These recommendations are based on the ABCD guidelines on SARS-CoV-2 (as of 30 December 2020) and will be updated regularly on the ABCD homepage (www.abcdcatsvets.org).

**What is the risk of a SARS-CoV-2-positive person infecting his or her cat?**

To date, only few cats have been identified as infected following contact with SARS-CoV-2-positive people. To reduce the risk of infection, close contact with pet cats should be avoided in households where people are infected with SARS-CoV-2 or have symptoms of COVID-19. However, cats should not be rehomed or relinquished.

**What is the risk of a SARS-CoV-2 positive cat infecting his or her owner?**

At the time of writing, no transmission event from cats to humans has been reported. More than 80 million human COVID-19 cases have been reported worldwide and human-to-human transmission is the main route of infection.

**What are the clinical signs linked to SARS-CoV-2 infection in cats?**

Following experimental infection, infectious virus was found in the upper respiratory tract, so respiratory signs might develop, or infection could be subclinical. Kittens were more susceptible following experimental infection compared to young adult cats, and infectious virus was isolated from the intestines of experimentally infected kittens, but not from young adult cats. The clinical signs that might be seen include respiratory signs, enteric signs, or infections might be subclinical in some animals.

**What should (self-isolating) SARS-CoV-2-positive people do if they have a cat?**

Contacts between COVID-19 patients and their pets should be limited to a minimum. However, cats in such households should remain in their home. It is preferable that they are looked after by another asymptomatic member of the household.

**What should be done with the cat if the owner needs to go to hospital?**

If the patient lives alone or needs to be hospitalised, the cat should remain at home and be cared for by friends or family of the patient, observing strict hygiene measures upon entering/leaving the home. It is not recommended to rehome, isolate, or even euthanise cats in these circumstances.

**Can cats carry the virus on their fur and should they be disinfected?**

Although it has not been proven, it is possible that fomite transmission could occur via pets. Cats themselves should not be disinfected, only inanimate materials. However, strict hygiene should be observed by the owners (washing hands after contact with cats) and close physical contact with cats (e.g., licking face, sharing food, or towels) should be avoided.

**Should vets be testing such cats routinely?**

The testing of cats for SARS-CoV-2 infection is not recommended; tests and reagents must be prioritised for human testing.

**What measures should cat owners take in COVID-19 affected areas?**

There is a risk of cats contracting SARS-CoV-2 from their owner, but the risk is minimised if standard hygiene measures are observed: avoid too close contact (e.g., licking face, sharing food, or towels), washing their hands with soap and water for at least 20 s (the WHO recommends even 40 s) before and after being near or handling their animals, and regularly cleaning the litter box. This will minimise the risk of any zoonotic diseases.
